# Exact distributed kinetic Monte Carlo simulations for on-lattice chemical kinetics: lessons learnt from medium- and large-scale benchmarks

**DOI:** 10.1098/rsta.2022.0235

**Published:** 2023-07-10

**Authors:** Giannis D. Savva, Raz L. Benson, Ilektra-Athanasia Christidi, Michail Stamatakis

**Affiliations:** ^1^ Thomas Young Centre and Department of Chemical Engineering, University College London, Torrington Place, London WC1E 7JE, UK; ^2^ Research Software Development Group, Advanced Research Computing Centre, University College London, Gower Street, London WC1E 6BT, UK

**Keywords:** kinetic Monte Carlo, lattice, time-warp algorithm, catalysis, materials science, distributed simulation

## Abstract

Kinetic Monte Carlo (KMC) simulations have been instrumental in multiscale catalysis studies, enabling the elucidation of the complex dynamics of heterogeneous catalysts and the prediction of macroscopic performance metrics, such as activity and selectivity. However, the accessible length- and time-scales have been a limiting factor in such simulations. For instance, handling lattices containing millions of sites with ‘traditional’ sequential KMC implementations is prohibitive owing to large memory requirements and long simulation times. We have recently established an approach for exact, distributed, lattice-based simulations of catalytic kinetics which couples the Time-Warp algorithm with the Graph-Theoretical KMC framework, enabling the handling of complex adsorbate lateral interactions and reaction events within large lattices. In this work, we develop a lattice-based variant of the Brusselator system, a prototype chemical oscillator pioneered by Prigogine and Lefever in the late 60s, to benchmark and demonstrate our approach. This system can form spiral wave patterns, which would be computationally intractable with sequential KMC, while our distributed KMC approach can simulate such patterns 15 and 36 times faster with 625 and 1600 processors, respectively. The medium- and large-scale benchmarks thus conducted, demonstrate the robustness of the approach, and reveal computational bottlenecks that could be targeted in further development efforts.

This article is part of a discussion meeting issue ‘Supercomputing simulations of advanced materials’.

## 1. Introduction

Kinetic Monte Carlo (KMC) simulations have made a significant contribution towards understanding and predicting the dynamic properties of materials [1–4]. Among the different fields, heterogeneous catalysis has widely adopted lattice-based KMC simulations, whereby the catalytic surface is represented by a set of connected sites, on which adsorbates can bind and react. Crucially, the rate constants of the pertinent elementary reaction events, i.e. adsorption/desorption, surface site hopping (diffusion), and reaction, can be calculated by lower-level methods, e.g. *ab initio* quantum chemistry methods or molecular dynamics. Combining KMC with these methods grants it significant predictive power and makes it the method of choice for unravelling the complexity of catalytic kinetics. The increasing popularity of lattice-based KMC is evidenced by the number of mature software codes available that implement this method. Some examples of widely adopted such codes include *Zacros* [5–10], SPPARKS [11,12], KMCLib [13] and kmos [14,15].

While increasingly popular, KMC simulations can be computationally challenging. The cost of a KMC simulation is, in principle, determined by the complexity of the chemical system under study in terms of number of reactions and adsorbate–adsorbate lateral interactions (either short- or long-range). For the most complex chemical reaction systems, KMC runs can require wall times ranging from several hours to several days for a sufficient number of elementary events to be executed, and accurate statistics to be obtained. A key computational limitation is the serial (sequential) nature of KMC [16], whereby execution of an event, e.g. an adsorption on a certain site, depends on the previous history, e.g. past events that result in that adsorption site being empty. Events must therefore be executed sequentially to ensure that the simulated history is self-consistent. This serial execution has limited the applicability of ‘traditional' KMC algorithms to small domains, on the order of a few tens of nm [17,18].

Broadening the applicability of KMC to larger systems and improving its computational efficiency is an active area of research, and tends to focus on tackling specific needs, for example, addressing the timescale disparity [19–21], or on generic algorithms and implementations that improve certain procedures without affecting the accuracy (e.g. [6,9,22–24]). Despite these developments, KMC simulations on larger lattices, with millions of sites, have remained generally intractable.

Such large simulations may indeed be necessary to capture phenomena like spatio-temporal pattern formation and oscillations exhibited by reactions taking place in extended media. Pertinent research efforts are motivated by the need to unravel the complexity of such systems (i.e. obtain fundamental understanding), but also by the potential benefits of controlling, improving, and/or engineering novel chemical systems for technologies of practical interest. For instance, CO oxidation on Pt catalysts in surface science experiments [25] and in reactors [26–28], partial oxidation of methane on Pd catalysts [29], N_2_O decomposition on Cu-ZSM5 [30], and the NO reduction by CO, H_2_ or NH_3_ on Pt surfaces [31,32] have all been shown to exhibit oscillations that may markedly affect activity or selectivity. In general, it has been argued that oscillations and spatio-temporal pattern formation may lead to potentially dangerous operating regimes in industrial reactors, but when properly managed and effectively controlled they could lead to enhancements in the conversion [33], thereby motivating fundamental studies to better understand such phenomena. Moreover, patterns such as spiral waves have been observed in extended chemical media, a well-known case being the Belousov–Zhabotinsky reaction [34]. Applications of such spatio-temporal complexity that might be of practical interest encompass the development of chemical systems that can perform computations [35] or the control of biological systems [36].

When it comes to computational investigations of such phenomena with KMC, the size of the lattice that is required to capture the dynamics of interest is dictated by the characteristic wavelength of the pattern. For example, the spiral waves reported by Nettesheim and co-workers for the catalytic CO oxidation on Pt(110) [25], the wavelengths are on the order of a few micrometres, requiring tens of millions of sites to be reproduced. Since the serially executed KMC implementations are unable to simulate systems in which patterns form on the micrometre scale, other means must be sought to make such simulations possible.

This has motivated the development of approaches in which the computational workload is distributed to multiple processing units (computer processors), thereby enabling the execution of KMC simulations in parallel [37–42]. Conceptually, these methods involve domain decomposition and algorithmic protocols to execute elementary events concurrently, in such a way that (eventually) the simulated history is self-consistent, i.e. free of conflicts due to events occurring on the boundaries of the subdomains. This self-consistency is achieved either via synchronization or by rolling back in time and correcting (re-simulating) the history that is invalidated because of the boundary conflicts. In the latter approach, each processing unit (PU) generates a KMC trajectory for its subdomain and once all those trajectories are validated as consistent up to a specific point in time, they are all ‘registered' to the global history that corresponds to the entire computational lattice.

Owing to the technical complexities of such distributed KMC simulation approaches, software implementations thereof in the fields of catalysis and materials science are currently scarce. For instance, SPPARKS [11,12] has implemented the approximate semi-rigorous synchronous sublattice algorithm [39] and SPOCK [43] includes an exact parallel KMC implementation based on the Time-Warp paradigm [37]. Still though, the latter implementation is not generic, and the user needs to provide system-specific code when building custom models. Also, both approaches mentioned above, lack validation procedures that would verify the correctness of the implementation.

To address these challenges and deliver a generic, parallel KMC implementation that can be validated, Stamatakis and co-workers have coupled the optimistic Time-Warp algorithm with the graph-theoretical KMC framework into the software package *Zacros* [5]. Preliminary benchmarks have demonstrated acceleration factors of more than 3 orders of magnitude on 400 processing units for a simple system with adsorption/desorption and up to first nearest-neighbour lateral interactions [5]. The better-than-expected performance was attributed to the smaller memory requirements and faster memory reading and writing, in addition to distributing the time-consuming operations to several processing units. A more complex system was also benchmarked in [5], which includes 22 elementary events capturing CO oxidation dynamics on a Pd(111) lattice with two site types (fcc and hcp), and incorporates a cluster expansion with 88 patterns, including single-, two- and three-body patterns [44]. For this system, a speed-up factor of about 110 × was obtained when simulating a lattice with more than 13.4 million sites on 729 processing units, compared with a serial run.

While demonstrating the power of the new approach coupling the Time-Warp algorithm with the graph-theoretical KMC framework, all these benchmarks entailed systems in which the adsorbate layer is homogeneous on the nanometre scale and beyond. Indeed, while local site-to-site correlations can be observed, e.g. (2 × 2) or (√3 × √3) ordering, no large-scale concentration gradients are exhibited by these systems. Since, as argued earlier, pattern formation is of particular interest in chemical kinetics and catalysis, this work focuses on benchmarks of our approach on a prototype system capable of exhibiting spiral wave pattern formation. This system is a lattice-based variant of the Brusselator system invented by Prigogine and Lefever to study non-equilibrium instabilities [45–47]. We demonstrate that our distributed KMC algorithm is capable of reproducing the spiral wave patterns of this system, and we explore the robustness and computational efficiency of the approach.

The rest of this paper is organized as follows. First, we provide a brief overview of the Time-Warp algorithm. Then, we provide a detailed description of the lattice benchmark model. Finally, we present our results and discuss some technical and computational aspects regarding the observed performance.

## 2. Methodology

### (a) Overview of the Time-Warp algorithm

The concept of Virtual Time and the Time-Warp algorithm are thoroughly discussed in the original paper by Jefferson [37], and the full details of our implementation coupling this algorithm with the graph-theoretical KMC can be found in [5]. Below, we provide a brief overview thereof.

Based on the Time-Warp paradigm, the main idea behind distributed KMC simulation is to decompose the entire lattice domain into smaller, non-overlapping, subdomains, each one of which is assigned to a different PU (also referred to as a *processing element* (PE)). A PU (or PE) is conventionally a CPU core. Each PU stores and deals with not only the lattice state of the subdomain to which it has been assigned, but also a ‘halo' region comprising sites from neighbouring subdomains. The depth of this halo region is chosen to be just large enough to handle all possible boundary events, by considering the spatial extent of all the adsorbates (which may be multi-dentate), elementary reactions and lateral energetic interactions involved [5]. In general, a halo region with a small area relative to that of each subdomain is associated with good parallel efficiency, since each PU executes the usual KMC method independently from other PUs for events within the ‘private portion' of its subdomain. The latter encompasses sites that are essentially far enough from shared boundaries that they do not immediately affect events happening in other domains. However, events that occur close to such boundaries, i.e. within one or more halo regions, necessitate some ‘collaboration' between the PUs sharing theseboundaries.

Thus, all events that happen away from the boundaries are executed concurrently by each PU, whereas the events at the boundaries are communicated among PUs and handled appropriately. Owing to the asynchronous nature of the execution of events and the random time advancement in KMC simulations, each PU has its own simulation time; hence, causality violations may occur, as will be discussed in more detail shortly. For the KMC trajectory to be exact, these violations need to be resolved in an algorithmically robust manner. The Time-Warp algorithm provides the required ‘machinery' so that exact KMC simulations can be performed in a distributed manner.

**Figure 1.**
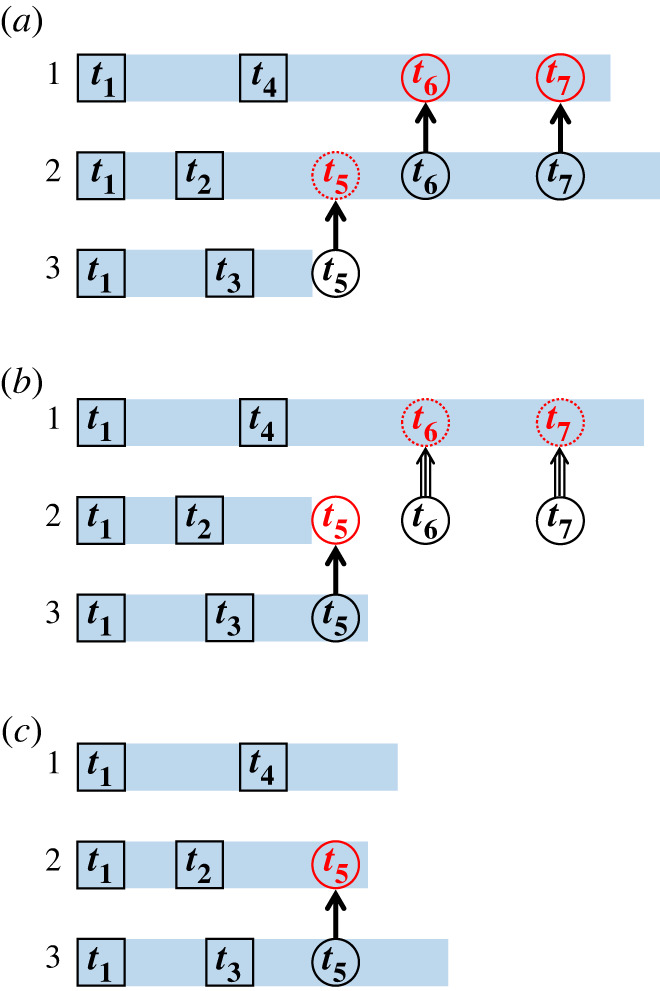
Schematic procedure of causality violations and of the way they are resolved. The blue rectangular bars represent KMC timelines; black squares represent snapshots saved; circles with inward and outward arrows represent received and sent messages, respectively; triple arrows represent anti-messages. (*a*) PU_2_ receives a message with timestamp *t*_5_, which is at its past. (*b*) PU_2_ reverts back to time *t*_2_ using a saved snapshot and re*-*simulates the history until *t*_5_. PU_2_ sends anti-messages with timestamps *t*_6_ and *t*_7_ corresponding to the previously sent messages. (*c*) PU_1_ receives the anti-messages and reverts back to *t*_4_ using a saved snapshot. (Online version in colour.)

Consider a scenario in which process PU_1_ executes an event at a boundary shared with its neighbour PU_2_ (figure 1 for a visual representation). The latter PU needs to have knowledge about this event, and thus, an appropriate message is sent from PU_1_ to PU_2_. If the receiving processor, PU_2_, is lagging behind PU_1_, then handling the message is straightforward. The message is put into a queue and appropriate action is taken when PU_2_ reaches that particular KMC time. What is less straightforward, however, is when the sender, PU_1_ lags behind the receiver, PU_2_. To better appreciate this, suppose that the message about an event that had just occurred on the timeline of PU_1_ is communicated to PU_2_. That message has a timestamp of *t*_1_. However, PU_2_ is ahead (*t*_2_ > *t*_1_) and therefore the message pertains to its past. The history of PU_2_ from time *t*_1_ onwards is wrong because the communicated event has not been accounted for; this constitutes a *causality violation*.

The only way to resolve this causality violation is to revert (roll back) to a time before *t*_1_ and re-simulate the history, taking into account the communicated event. To enable such rollbacks, all PUs need to have snapshots of the system available along their history, so that they can revert to them when needed. Therefore, as each PU propagates only its allocated subdomain, it takes complete snapshots of its entire simulation state and stores them in memory at regular intervals during the course of the KMC simulation, e.g. every 1000 KMC steps.

The situation described above is even more complicated if PU_2_ had sent messages to other units, after KMC time *t*_1_ but before the causality violation occurred. Since the history of PU_2_ between *t*_1_ and *t*_2_ is now being re-simulated, any actions performed by other PUs as a result of such messages need to be corrected. Then, PU_2_ needs to send each of those units an ‘anti-message', that encodes an ‘undo’ action to a previously sent message. The PUs that receive these anti-messages may have to revert back as well and correct their histories (this would happen if the timestamp of an anti-message is smaller than the current KMC time of the receiving PU). A schematic of causality violations involving three processing units is illustrated in figure 1. It is possible that the causality violations and the rollback procedure produce a cascade of violations that could affect the entire domain and slow down the whole simulation. However, such cases are system-dependent and are rare in practice. In any case, the Time-Warp algorithm provides all the procedures necessary to resolve such conflicts and ensure the consistency of the simulated history.

As already noted, rolling back in KMC history necessitates that PUs regularly save snapshots of the entire state of the simulation in the memory. However, since the latter is limited, the simulation needs a procedure to free up memory and delete the snapshots that are no longer needed. Such a procedure involves global communications. Therefore, at regular intervals, all PUs communicate with each other to determine which one has the smallest KMC time, *t*_s_. Assuming that there are no pending messages, the histories of all PUs are mutually consistent up until *t*_s_, and thus, any snapshots taken before *t*_s_ may be safely deleted. If there are pending messages, the smallest between the PUs’ KMC times and the timestamps of pending messages is taken as *t*_s_. The latter (*t*_s_) is referred to as Global Virtual Time (GVT) and represents the time-point up to which the global simulation history is consistent and will never again be revised (corrected). Thus, the GVT serves as a metric to quantify the overall progress of the KMC simulation and decide when the simulation can be terminated (i.e. when the GVT exceeds the final simulation time set by the user).

An important detail regarding the Time-Warp implementation pertains to the case in which all the available memory is filled up before a global communication takes place to remove the no-longer-needed snapshots. In this case, certain procedures must be invoked to: **(a)** sparsify the queue in which snapshots are saved, e.g. by deleting every other snapshot to free memory, and **(b)** increase the interval over which snapshots are taken so that the queue does not fill up again after the GVT is calculated (e.g. instead of saving a snapshot every 1000 KMC steps, now save every 2000 KMC steps). If needed, the above procedures are invoked again to ensure that the frequency of saving snapshots is appropriate, given the available memory.

The Time-Warp algorithm, as briefly introduced above, includes two user-defined parameters that affect the computational performance. These are **(a)** the number of KMC steps after which the PUs take a snapshot and **(b)** the real time interval after which the global communication takes place. The investigation of the impact of these parameters on the performance of the Time-Warp algorithm is, however, out of the scope of the current study. For this reason, in our simulations we have chosen optimal values following a limited number of shorter runs and we kept them fixed for all the runs presented in the following section.

### (b) Lattice-based Brusselator benchmark system

The Brusselator reaction mechanism was introduced by Prigogine and Lefever in the late 60s to study symmetry-breaking instabilities in dissipative systems [46]. It involves two main species, one of which promotes its own production in an autocatalytic manner. This results in rich dynamic behaviour, specifically oscillations, under certain parametric constraints and assuming fast diffusion (well-mixed system). Furthermore, if the reaction is embedded into a spatially extended medium into which molecular species can diffuse (in addition to reacting), an instability occurs when diffusion is slow compared with reaction, and as a result, the system can exhibit spatio-temporal pattern formation. Such a system is therefore ideal for demonstrating the capabilities of our distributed KMC approach by reproducing such spatio-temporal patterns at large scales.

To use our KMC approach on the Brusselator system, the reaction mechanism of the latter needs to be adapted for on-lattice simulation. The pertinent elementary reactions (excluding diffusion events) are shown in table 1, vis-à-vis those of the original system [46]. In the surface events, a star, *, denotes an empty site, and a species with a star, e.g. Y*, represents an adsorbed molecule. Thus, in the lattice-based Brusselator, two reactions that produce and consume species X in the ‘original' Brusselator are lumped into an adsorption/desorption event. The trimolecular step is simulated with Y* in the middle, surrounded by two X* molecules. The conversion of X* into Y* is considered as a reversible Eley–Rideal reaction with B and D as gas-phase species. The reason that this reaction was taken to be reversible in our model, is to avoid getting trapped into a state in which the lattice is completely covered by Y* species. In such a case, if the reaction in discussion was irreversible, there would be no mechanism for X* molecules to reappear on the surface: X would no longer be able to adsorb, since there would be no empty sites, and there would be no mechanism to convert Y* into X*; hence, the surface would be poisoned. This is not a concern in the ‘original' Brusselator, in which there are no constraints on the molecule numbers (or species concentrations) and thus reaction (a) of table 1, which brings X* into the system, never ceases. Furthermore, we neglect species A and E of the ‘original' Brusselator, without distorting the dynamics of the reaction mechanism, since anyway the concentrations of both of these species were kept constant in the analysis of Prigogine & Lefever [46]. In our lattice-based Brusselator, defining the kinetic and thermodynamic constants for adsorption (the latter being the ratio between the kinetic constants of adsorption versus desorption) enables us to precisely tune the fluxes of X into and out of the system. Moreover, the partial pressures of B_(gas)_ and D_(gas)_ are kept constant, in line with the concentrations of species B and D of the ‘original' Brusselator being maintained at constant levels.

**Table 1.** Elementary events (not including diffusions) of the lattice-based Brusselator adaptation, vis-à-vis those of the original system. The labelling of the events corresponds to that in [36].

event label	‘Original’ Brusselator	lattice-based Brusselator
(a, d)	A→X,X→E	$X_{\left(\mathrm{gas}\right)}+*↔X^{*}$
(b)	2X+Y→3X	$2X^{*}+Y^{*}\to 3X^{*}$
(c)	B+X→Y+D	$B_{\left(\mathrm{gas}\right)}+X^{*}↔Y^{*}+D_{\left(\mathrm{gas}\right)}$

The spatially extended medium onto which the reactions are happening is represented as a two-dimensional fourfold (square) lattice. To ensure effective adlayer mixing at a local level, three diffusion steps were considered: diffusion of X*, as well as Y*, to a neighbouring vacant site, but also exchange between X* and Y* (last three rows of table 2). The diffusion of Y* was taken to be 100 times slower than the other two diffusion events, in order to induce the instability necessary for spiral wave formation. The activation energies of all events were taken to be zero and there are no adsorbate lateral interactions in this model. Thus, the rate constants of surface events were equal to the corresponding pre-exponential (*A*_fwd_ or *A*_rev_), and for events involving gas-phase species, the pre-exponentials were multiplied by the corresponding partial pressures. For instance, for the adsorption step (i)-forward, the rate constant is 0.7·P_X(gas)_ while for the desorption it is 0.7/0.91 = 0.769 s^–1^. The gas species partial pressures were: P_X(gas)_ = 0.4 bar, P_B(gas)_ = 0.4 bar, P_D(gas)_ = 0.2 bar, and the rate constants calculated with these values are shown in table 2. Finally, note that in the latter table, step (ii) is depicted with an angle of 90° but it can also occur when the three sites (occupied by the Y* and two X* adsorbates) are co-linear. A detailed discussion on the preliminary investigations that led to the choice of the parameter values just noted, appears in the electronic supplementarymaterial.

**Table 2.** Schematics and values of the pre-exponentials of the elementary events of the lattice-based Brusselator. The rate constants are also shown for each event at the conditions of the simulations; these rate constants give the expected rate of occurrence of each event per instance of the pertinent coverage pattern on the lattice.

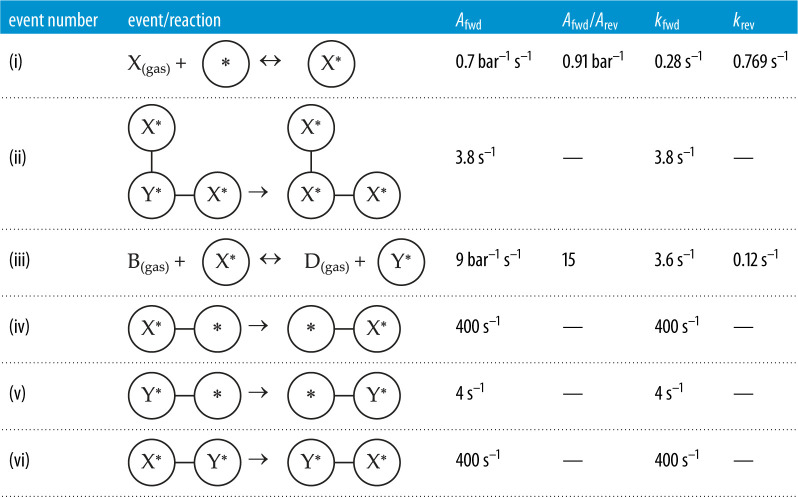

## 3. Results and discussion

**Figure 2.**
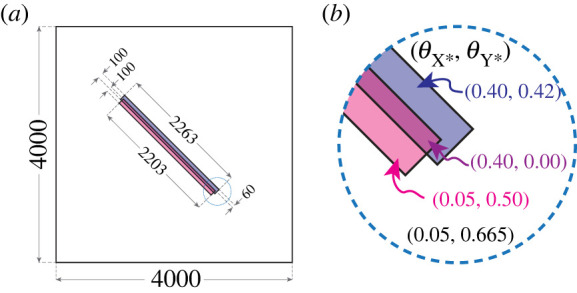
Geometry and coverages used as initial condition. (*a*) The overall domain of 4000 × 4000 sites was separated into four sections using two overlapping rectangles as indicated in the diagram. The centre of the larger rectangle is positioned exactly at the centre of the domain, and the long sides of both rectangles form an angle of 45° with the side of the overall domain. (*b*) Adsorbed molecules of species X* and Y* were seeded onto the lattice thereby achieving the coverages denoted in the diagram, for each of the four sections. (Online version in colour.)

We now proceed to discuss the results of a lattice-based Brusselator simulation in which the evolution of spiral wave patterns was observed from an appropriate initial state (initial condition). This simulation was performed with the Time-Warp algorithm and the graph-theoretical KMC approach as implemented in our KMC software *Zacros*. A domain of size 4000 × 4000 was used (16 million sites in total) and the simulation started from an initial state in which molecules of species X* and Y* were seeded throughout the domain in such a way as to invoke the formation of spiral waves (figure 2), by creating a ‘broken' wavefront that curls around its edges in opposite directions. The mechanism of wave propagation is discussed in detail below, but we can already note that at the front-end of the wave, the ‘activator’ species X* exhibits a sharp increase in coverage, which propagates through the medium as Y* gets converted into X*. This peak coincides with a drop of Y* coverage, while this species is gradually repleted at the rear-end of the wave. Imposing solely a higher initial coverage of X* in a linear segment of the lattice would result in an oval wave, since conversion of Y* would be possible in all directions. To create a ‘broken' wavefront, we additionally impose low coverage of Y* (pink shaded domain in figure 2) so that the wave can propagate only towards the upper right corner of the domain (since there is not enough Y* to get converted at the opposite direction), and then curl spontaneously around at its edges. Some preliminary simulations in smaller domains helped in adjusting the thicknesses of the domains and identifying minimum lengths for which prominent spiral patterns would emerge.

**Figure 3.**
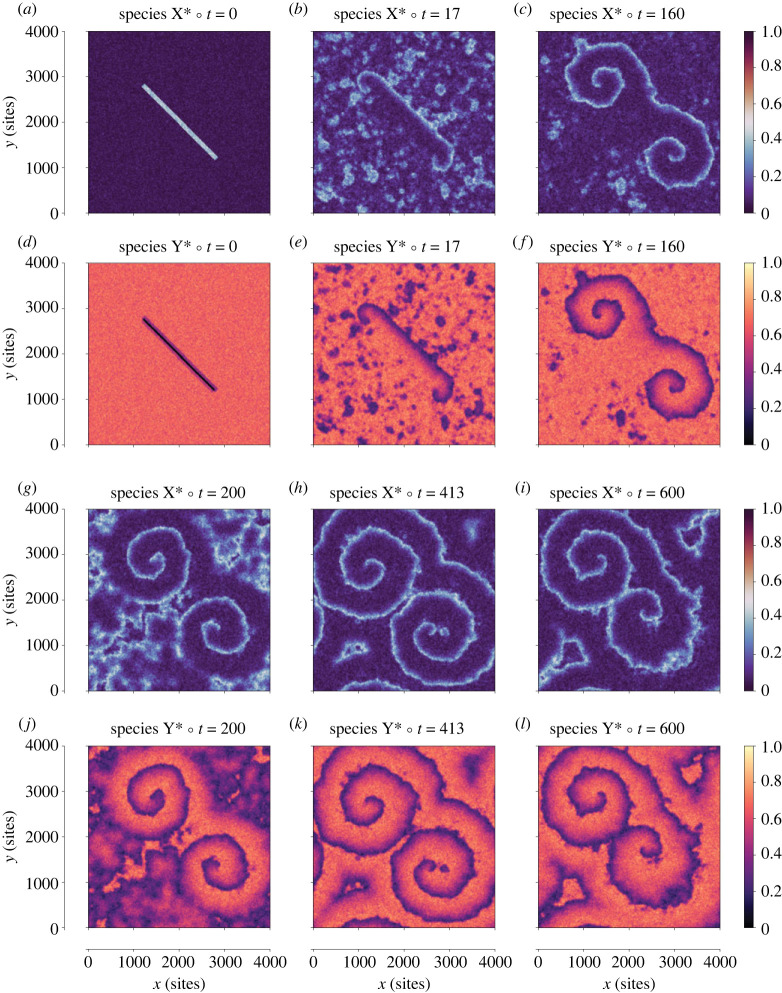
Snapshots of the average fractional coverages of X* (*a, b, c, g, h, i*) and Y* (*d, e, f, j, k, l*) at various times (in units of seconds) during the simulation. The coverage of X* spans a range of about 0–0.5 and that of Y* a range of about 0.3–0.7. The initial state (*a, d*) leads to the formation of two spirals rotating in opposite directions. At time 413 s, a secondary wavefront emerges close to the tip of the lower spiral, and eventually pushes the tip closer to the centre of the domain. (Online version in colour.)

**Figure 4.**
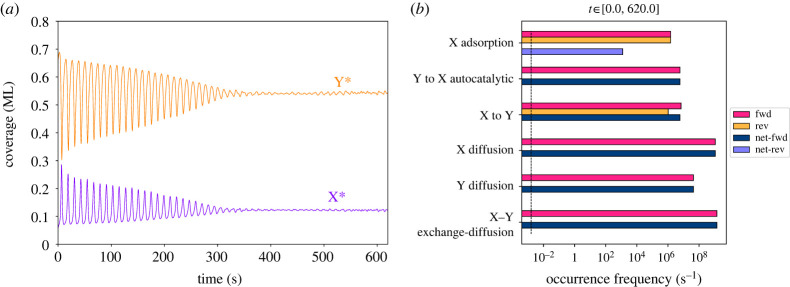
(*a*) Fractional surface coverages of X* and Y*. (*b*) Frequency of elementary events executed, given in number of events per unit time for the overall lattice. (Online version in colour.)

Representative snapshots of the average coverages of the two species over time are shown in figure 3. The initial state of figure 2, also shown at *t* = 0 s in figure 3*a,d*, gives rise to two spirals that rotate in opposite directions. To explain how these patterns emerge, we first revisit the dynamics of the underlying reaction network from a qualitative standpoint, noting that the key feature of the Brusselator is the competition between the rapid conversion of Y* into X* in the presence of pre-existing X*, and the slower conversion of X* back into Y*. More specifically, the first conversion is achieved via the autocatalytic reaction (ii) of table 2, which requires *two* molecules of X* to be located at neighbouring sites with Y*, resulting in a quadratic rate-versus-coverage dependence. The latter dependence leads to threshold behaviour: for low coverages of X* the reaction rate is negligible, while, for higher coverages, the rate rises sharply. Of course, the rate also depends on the coverage of Y*, so when this species gets depleted, the rate of conversion of Y* to X* drops dramatically. At that point, the system relies on the forward step of reaction (iii) of table 2 for Y* to be replenished. This reaction, however, exhibits first order kinetics with respect to the coverage of X* and therefore proceeds relatively slowly. Consequently, the Brusselator exhibits oscillations in which the coverage of X* rises rapidly and concurrently with a depletion of Y* (‘activation' phase) followed by a more gradual or delayed repletion of Y* (‘refractory' phase). Such oscillations are indeed observed during the initial times of the simulation, as shown in figure 4*a*.

In the spatially extended medium simulated, these oscillatory dynamics in tandem with the diffusion of the two interacting species play a key role in the formation of wavefronts that propagate through the medium. Crucially, the diffusion rate of the activator species X* needs to be higher than that of Y*, so that any gradients in the coverage of the latter persist for sufficiently long timescales, enabling the wavefront to propagate effectively towards regions with high Y* coverages. Thus, in the absence of any external influence, the inherent noise of the KMC simulation leads to the emergence of wavefronts from random points in the lattice. These can be clearly seen in figure 3*b,e*, in the areas far beyond the rectangles imposed in the initial state as a perturbation. The latter led purposely into a situation in which a ‘broken' wavefront started rotating around its tip, a behaviour known as ‘re-entry' in the study of chemical waves and excitable systems [48], which eventually leads to the formation of the two spiral waves. The range of these spirals extends progressively, which also leads to the attenuation of the oscillations of figure 4*a* due to the averaging-out of the species coverages over the spatial coordinates. Eventually, at around 400 s, the spiral patterns take up the entire domain (figure 3*h,k*). The time to form the fully developed spirals depends mainly on the period of the rotation of the spiral and the size of the lattice: at every rotation of the spiral the pattern expands slightly, because the period of rotation is slightly shorter than that of the oscillation. In our simulation, it took about 27–28 spiral rotations to reach a fully developed at time around 300 s (for comparison, during this time interval, regions far from the spiral would have exhibited only about 25 oscillation peaks). Larger lattices would require longer times for the spirals to take over the entire domain, and faster rotations would result in shorter times for this to happen.

While the patterns are robust, they are subject to perturbations due to noise (stochasticity); for instance, at time around 413 s, a secondary wavefront emerges close to the tip of the lower spiral and pushes the tip slightly closer to the centre of the domain (figure 3*i,j*). In the electronic supplementary material, we plot spatial reactivity maps within a short time interval at that time, showing the location where each of the elementary events of table 2 took place. The overall statistics, averaged in space and time appear in figure 4*b*.

Next, we focus on some technical (computational) aspects of these KMC runs. Owing to the large size of our lattice, the simulation was distributed over 25 × 25 = 625 PUs, so that each one of them is assigned a subdomain of 160 × 160 sites. The entire simulation was run on Thomas, the UK National Tier 2 High Performance Computing (HPC) Hub in Materials and Molecular Modelling. The computational nodes on Thomas each contain 24 CPU cores (2 × 12-core Intel(R) Xeon(R) E5-2650 v4) and 128 GB RAM. Because of the wall time restrictions, this simulation was broken into ‘chunks’ of 24 or 48 h each. *Zacros*'s core implementation provides a functionality for stopping and resuming a simulation, and consequently, runs that use this checkpointing feature produce identical results with continuous runs, while being more robust against system faults. The simulation reached an overall KMC time of about 620 KMC seconds and involved more than 1.6 trillion elementary events (though, due to the rollbacks of the Time-Warp algorithm, the actual times and number of events executed by the PUs were larger, as will be discussed later). In terms of real time, the distributed simulation was running for 38 days.

**Figure 5.**
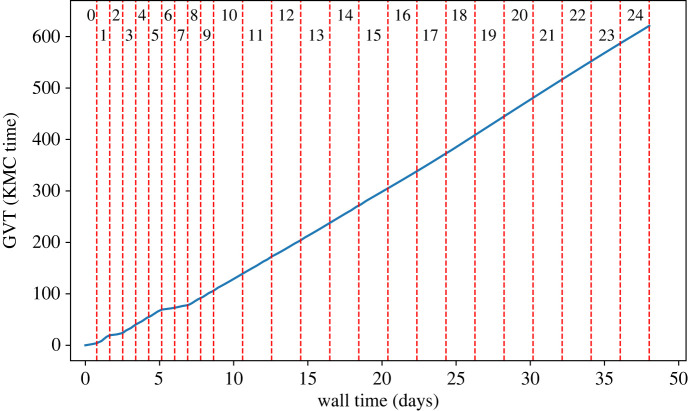
Global virtual time (GVT) plotted against wall time for our Brusselator simulation with 625 processors. Red dashed lines are used to indicate where the simulation was paused and later restarted. The numbers on the top represent the simulation restart index of each chunk. (Online version in colour.)

Figure 5 illustrates the overall progression of the simulation. More specifically, we plot the GVT against the wall time for the entirety of the simulation, with red dashed lines used to indicate where the simulation was paused and later resumed. Each chunk is also numbered using its restart index shown at the top of figure 5 (restart index of 0 denotes the initial run). As explained above, the GVT is to be interpreted here as a metric to quantify the progression of the overall KMC simulation, keeping in mind, of course, that the subdomains evolve asynchronously. For most of the simulation chunks, the distributed simulation has a consistent progression rate. This, however, is not the case for the early stages of the simulation. Up to the seventh chunk, the GVT appears to be progressing with a fluctuating rate. At first glance, one might attribute this behaviour to the evolving nature of the adsorbate layer as the spirals began to develop from the initial conditions. Yet, upon closer inspection, one notices that the GVT advancement rate within each chunk is almost linear. In addition, simulation chunks with restart indices 2, 6 and 7 have a suspiciously low GVT advancement rate that does not appear to correlate with the dynamics of our benchmark system.

**Figure 6.**
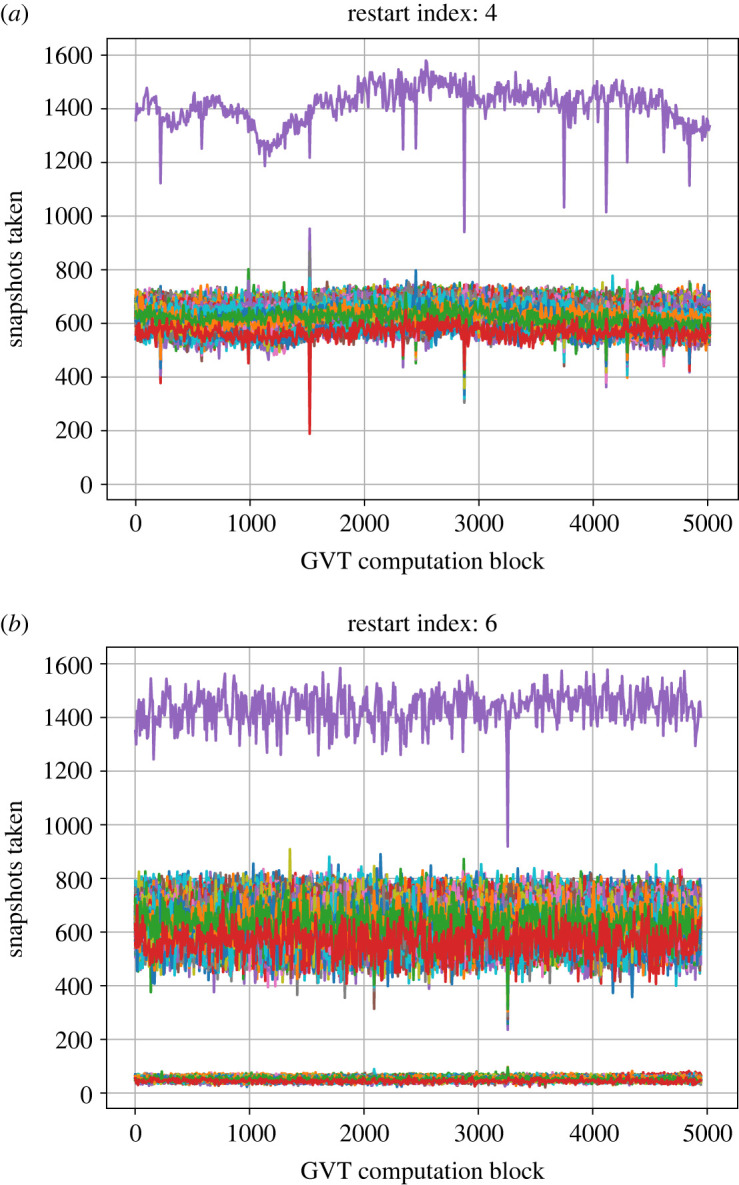
Number of snapshots taken plotted against the GVT computation block for (*a*) restart number 4 and (*b*) restart number 6 of the simulation. Each curve represents one PU so there are 625 curves in each of the panels (*a*) and (*b*). In (*a*) all of the PUs except one save on average the same number of snapshots. In (*b*), there are several PUs that save considerably fewer snapshots for the whole duration of the simulation. The computational load imbalance is responsible for the single PU that shows a higher execution of KMC steps in both panels. (Online version in colour.)

To further investigate this behaviour, we examine the number of snapshots taken during the interval between two global communications. The latter interval is also termed as ‘GVT computation block'. Since the snapshots are saved regularly by all PUs, i.e. at every fixed number of KMC steps, one may conclude that the PUs that save more snapshots execute more KMC steps, whereas the PUs that save fewer snapshots are executing fewer KMC steps. Figure 6 illustrates two such cases. In panel (a), that corresponds to the 4th simulation chunk (cf. figure 5), all but one of the 625 PUs are saving approximately the same number of snapshots during a GVT computation block. Having one fast-progressing PU was observed in all the runs, and this was attributed to the way processors from different nodes of the HPC cluster Thomas were allocated. In particular, since each node on Thomas had 24 processors, the allocation was done by utilizing all processors of 26 nodes and only one processor of an additional (27th) node; the latter processor was the fast-processing PU, and all other PUs exhibited slower but equal (on average) speeds. On the contrary, for the slowly progressing 6th simulation chunk (figure 6*b*), there is number of PUs that save considerably fewer snapshots per GVT computation block. Upon further investigation, it was identified that all of the slowly progressing PUs belonged to a limited number of computational nodes. This was an indication that either the hardware of the aforementioned nodes was not operating at full capacity or certain background processes were consuming computational resources. In subsequent chunks, the ‘problematic nodes’ were excluded and thus, we started observing a consistent pattern in the GVT progression (8th chunk onwards in figure 5).

**Figure 7.**
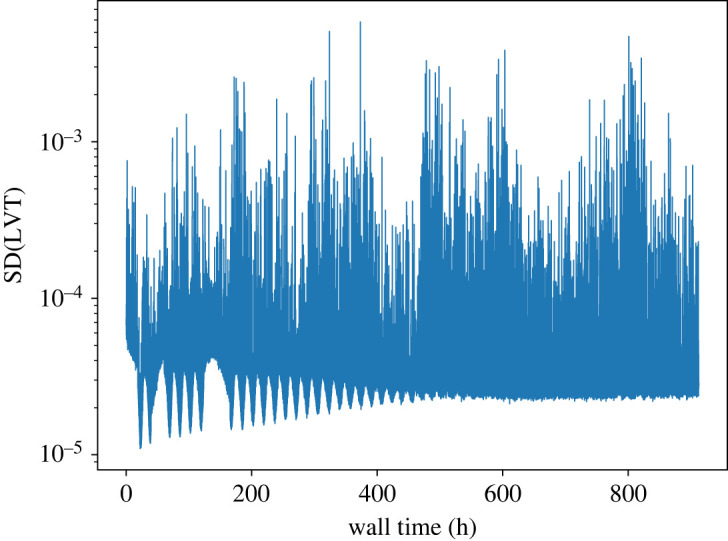
Standard deviation of the local virtual times (LVTs) plotted against wall time for our Brusselator simulation with 625 processors. (Online version in colour.)

It is pertinent to consider the extent to which, if any, the asynchronization of processors grows over time. Kolakowska & Novotny [49] among others [50–52] have applied techniques from non-equilibrium surface growth to understand the evolution of the virtual time horizon (VTH), defined as the collection of all local virtual (KMC) times (LVTs) in a parallel simulation. The degree of asynchronization is then analogous to the roughness of a one-dimensional surface, such that each LVT corresponds to the height of a column on the surface; the columns grow via pseudorandom ‘depositions’ of kinetic waiting times. The roughness of the VTH can be quantified by the standard deviation of the LVTs, expected to grow like √t [49]. In figure 7, we plot the progress of this quantity during our Brusselator simulation. While there are large fluctuations, it is not possible to identify any consistent growth. This is encouraging, since a higher degree of asynchronization may worsen simulation efficiency, but it is likely a fortuitous result of the frequent restarts in our simulation. In our Time-Warp implementation, each time the simulation is paused, all PUs roll back to the most recent KMC state that satisfies LVT < GVT; thus, only one snapshot is saved in the checkpointing file, thereby reducing disc space utilization. Hence the PUs become synchronized after every restart operation, effectively quenching any roughening of the VTH. We expect that roughening would be observed, however, in a longer, uninterrupted simulation. Pausing and resuming the simulation may thus be an effective way to mitigate VTH roughening in Time-Warp simulations, although further investigations are needed to verify this. Other methods have already been developed to suppress the roughening in synchronous (conservative) schemes for parallel discrete-event simulation [50], which might also be transferrable to asynchronous (optimistic) schemes such as Time-Warp.

While we did not observe asynchronization among the different processors, the simulation efficiency is hampered by the need to correct the simulated history via the inherent rollbacks of the Time-Warp algorithm. To quantify the pertinent overhead, we calculate the ratio between the KMC time that was rerun due to rollbacks versus the GVT advancement, for each interval between successive global communications. Clearly, this quantity is zero in the ideal case of decoupled domains because of the lack of any rollbacks. For the Brusselator simulation, this ratio is plotted in the electronic supplementary material, figure S1 for PUs 0, 100 and 624, for the last simulation chunk (index 24). For all PUs, the overhead is substantial, e.g. for PU 0, the ratio in discussion ranged between 5.8 and 69.2, and on average about 14.7 KMC time units had to be re-simulated for every KMC time unit of GVT advancement. For the fast-processing PU 624, this ratio was even higher, with a range between 13.0 and 122.2 and an average of 31.4.

To assess the potential of the distributed KMC algorithm, we executed a short serial run of the Brusselator system on the 4000 × 4000 lattice (using the sequential KMC algorithm on just one processor). This simulation was initialized with a lattice state that corresponded to the well-developed spiral patterns at *t* = 399.5. To enable meaningful comparisons among the different runs, we use the normalized simulation wall time, defined as the real-world time hours needed to simulate one KMC time unit. For the serial run on HPC cluster Thomas, the normalized simulation wall time was found to be 19.7 h. In comparison, for the distributed simulation over 625 PUs only 1.3 h of wall time are needed on average per KMC time unit. Therefore, the distributed algorithm achieves an acceleration factor of approximately 15 × and, most importantly, makes possible the simulation of our benchmark model within a reasonable amount of time. By comparison, the serially executed KMC simulation would need slightly more than 1.5 years of computation in order to obtain the same results (i.e. reach a final KMC time of 620 KMC time units) as those obtained by the distributed implementation in just 38 days.

Lastly, aiming to investigate the scalability and stability of the Time-Warp implementation in large-scale distributed runs, we performed a limited number of runs using the same benchmark set-up (Brusselator system on a 4000 × 4000 lattice) distributed over 1600 PUs instead of 625 PUs. The computational cluster Kathleen@UCL was used for these simulations, which is a UCL HPC Facility, comprising 192 discless compute nodes each containing 40 CPU cores (2 × 20-core Intel Xeon Gold 6248 2.5 GHz processors), 192 gigabytes of 2933 MHz DDR4 RAM, and an Intel OmniPath network. For the first chunk of this large run, the initial condition was that of the fully developed spirals at *t* = 399.5 (as in the serial run discussed in the previous paragraph). The simulation was run for 12 h and was restarted two more times to reach a final KMC time of 57.2 KMC time units in a total real time of 35 h. These runs revealed similar slowdown issues as those observed the runs with 625 PUs, due to slow hardware or background processes consuming computational resources (electronic supplementary material, figure S2). Yet, the acceleration factors achieved are significant. Table 3 summarizes both our medium- and large-scale distributed runs by presenting the computation cost of our simulations and the achieved acceleration factors by the distributed simulations as compared against the serial runs. As expected, distributing the workload over more PUs, namely over 1600 versus 625, we obtain a better (higher) acceleration factor of about 36 × .

**Table 3.** Normalized simulation wall time and acceleration factors of the distributed runs.

HPC cluster	simulation type	normalized simulation wall time (hours/KMC time)	acceleration factor
Thomas	serial	19.7	—
	distributed 625 PUs	1.3	15×
Kathleen	serial	15.6	—
	distributed 1600 PUs	0.44	36×

It is useful to note that the acceleration factor from the serial run to the distributed with 625 PUs is quite less than ideal due to the extra time required to save and delete state snapshots, perform rollbacks and re-simulate KMC history. As the number of PUs is increased further to 1600 and the subdomain area correspondingly decreases, the number of rollbacks (and fraction of time spent re-simulating history) will be even higher. However, the speed-up relative to 625 PUs is almost ideal (1600/625=2.56≈36/15=2.4). This may be explained by less time being required for saving/deleting snapshots, which are smaller and thus occupy less memoryper PU.

## 4. Conclusion

In this study, we demonstrated and benchmarked our coupled implementation of the Time-Warp algorithm [37] and the Graph-Theoretical KMC framework [7,8], for exact, distributed KMC simulations of reactive phenomena. The main idea behind the Time-Warp approach is to decompose the domain (lattice) into smaller subdomains and assign each one of them to a different PE. Events are executed ‘locally' for the internal sites of each subdomain, and events that involve boundary sites are communicated to the neighbouring subdomains. Since each subdomain has its own timeline, causality violations may occur when, for example, one subdomain that is ‘lagging behind' in KMC time, tries to send a species via diffusion to a nearby domain that is further ahead in time. The KMC history of the domain that receives the particle is no longer valid and needs to be corrected. This is achieved by saving snapshots along the KMC simulation and restoring the most suitable one when such conflicts arise. Corrections to the history are made repeatedly as necessary, up to the point that histories which are mutually consistent among all subdomains are obtained.

The key feature of this method is that it reproduces exactly the dynamics of the underlying stochastic model, without introducing numerical approximations (being only subject to the precision errors of the computing architecture used). This is particularly attractive when simulating complex systems in chemistry and catalysis, such as those that exhibit spatio-temporal inhomogeneities, with examples encompassing chemical oscillators in extended media (Belousov–Zhabotinsky reaction [34]) or pattern formation due to surface reconstruction in catalysis. For such simulations, the absence of artefacts due to numerical approximations is key to confidently ascertain the relationship between the underpinning reaction mechanisms and the observed behaviour.

To demonstrate our approach in this context, we developed a lattice-based variant of the Brusselator system and performed simulations for sufficiently large lattices and KMC times in which spiral wave patterns were successfully observed. The distributed runs were found to have overheads, stemming from the saving and restoring of snapshots, the necessary re-simulations to correct the history, and the communications among the PUs. Still though, the newly implemented parallelization in our software *Zacros* proved to be stable and performant, being able to simulate the Brusselator dynamics about 15 times faster when using 625 PUs, compared with the sequential KMC algorithm, and 36 times faster with 1600 PUs. Our approach is thus expected to be invaluable in future research efforts aiming at obtaining a fundamental understanding of intricate spatio-temporal phenomena on catalytic surfaces. Such phenomena could e.g. arise from surface reconstruction, which is considered responsible for pattern formation on even simple systems like CO oxidation on Pd(110) [25]. Harnessing the power of HPC architectures with our software could thus make it possible to capture new physics, and bridge the gap between the molecular scale and the meso- and macro-scales towards unravelling the complexity of heterogeneouscatalysts.

## Data Availability

The data are provided in electronic supplementary material [53].

## References

[1] Battaile CC. 2008 The kinetic Monte Carlo method: foundation, implementation, and application. Comput. Method Appl. M. **197**, 3386-3398. (10.1016/j.cma.2008.03.010)

[2] Ustinov EA, Do DD. 2012 Application of kinetic Monte Carlo method to equilibrium systems: vapour-liquid equilibria. J. Colloid Interf. Sci. **366**, 216-223. (10.1016/j.jcis.2011.09.074) 22014397

[3] Apostolopoulou M, Santos MS, Hamza M, Bui T, Economou IG, Stamatakis M, Striolo A. 2019 Quantifying pore width effects on diffusivity via a novel 3D stochastic approach with input from atomistic molecular dynamics simulations. J. Chem. Theory Comput. **15**, 6907-6922. (10.1021/acs.jctc.9b00776) 31603675

[4] Franco AA, Rucci A, Brandell D, Frayret C, Gaberscek M, Jankowski P, Johansson P. 2019 Boosting rechargeable batteries R&D by multiscale modeling: myth or reality? Chem. Rev. **119**, 4569-4627. (10.1021/acs.chemrev.8b00239) 30859816 PMC6460402

[5] Ravipati S, Savva GD, Christidi I-A, Guichard R, Nielsen J, Réocreux R, Stamatakis M. 2022 Coupling the time-warp algorithm with the graph-theoretical Kinetic Monte Carlo framework for distributed simulations of heterogeneous catalysts. Comput. Phys. Commun. **270**, 108148. (10.1016/j.cpc.2021.108148)

[6] Ravipati S, d'Avezac M, Nielsen J, Hetherington J, Stamatakis M. 2020 A caching scheme to accelerate kinetic Monte Carlo simulations of catalytic reactions. J. Phys. Chem. A **124**,7140-7154. (10.1021/acs.jpca.0c03571) 32786994

[7] Nielsen J, d'Avezac M, Hetherington J, Stamatakis M. 2013 Parallel kinetic Monte Carlo simulation framework incorporating accurate models of adsorbate lateral interactions. J. Chem. Phys. **139**, 224706. (10.1063/1.4840395) 24329081

[8] Stamatakis M, Vlachos DG. 2011 A graph-theoretical kinetic Monte Carlo framework for on-lattice chemical kinetics. J. Chem. Phys. **134**, 214115. (10.1063/1.3596751) 21663352

[9] Savva GD, Stamatakis M. 2020 Comparison of queueing data-structures for kinetic Monte Carlo simulations of heterogeneous catalysts. J. Phys. Chem. A. **124**, 7843-7856. (10.1021/acs.jpca.0c06871) 32870681

[10] Stamatakis M. 2013 *Zacros*: Advanced Lattice-KMC simulation Made Easy. See https://zacros.org

[11] Plimpton S *et al.* 2009 Crossing the mesoscale No-man's land via parallel kinetic Monte Carlo. Albuquerque, NM: Sandia National Laboratories. Report No.: SAND2009-6226.

[12] Plimpton S, Thompson A, Slepoy A. 2012 SPPARKS Kinetic Monte Carlo Simulator Sandia National Laboratories. See http://spparks.sandia.gov/.

[13] Leetmaa M, Skorodumova NV. 2014 KMCLib: A general framework for lattice kinetic Monte Carlo (KMC) simulations. Comput. Phys. Commun. **185**, 2340-2349. (10.1016/j.cpc.2014.04.017)

[14] Hoffmann MJ. 2014 kMC on steroids: a vigorous attempt to make lattice kinetic Monte Carlo modeling as fast as possible. See http://mhoffman.github.io/kmos/.

[15] Hoffmann MJ, Matera S, Reuter K. 2014 kmos: a lattice kinetic Monte Carlo framework. Comput. Phys. Commun. **185**, 2138-2150. (10.1016/j.cpc.2014.04.003)

[16] Papanikolaou KG, Stamatakis M. 2020 Chapter 7 - Toward the accurate modeling of the kinetics of surface reactions using the kinetic Monte Carlo method. In Frontiers of nanoscience (ed. P Grammatikopoulos), pp. 95-125. Amsterdam, The Netherlands: Elsevier.

[17] Pineda M, Stamatakis M. 2022 Kinetic Monte Carlo simulations for heterogeneous catalysis: fundamentals, current status, and challenges. J. Chem. Phys. **156**, 120902. (10.1063/5.0083251) 35364885

[18] Chutia A, Thetford A, Stamatakis M, Catlow CRA. 2020 A DFT and KMC based study on the mechanism of the water gas shift reaction on the Pd(100) surface. Phys. Chem. Chem. Phys. **22**, 3620-3632. (10.1039/c9cp05476f) 31995067

[19] Chatterjee A, Voter AF. 2010 Accurate acceleration of kinetic Monte Carlo simulations through the modification of rate constants. J. Chem. Phys. **132**, 194101. (10.1063/1.3409606) 20499945

[20] Danielson T, Sutton JE, Hin C, Savara A. 2017 SQERTSS: Dynamic rank based throttling of transition probabilities in kinetic Monte Carlo simulations. Comput. Phys. Commun. **219**, 149-163. (10.1016/j.cpc.2017.05.016)

[21] Dybeck EC, Plaisance CP, Neurock M. 2017 Generalized temporal acceleration scheme for kinetic Monte Carlo simulations of surface catalytic processes by scaling the rates of fast reactions. J. Chem. Theory Comput. **13**, 1525-1538. (10.1021/acs.jctc.6b00859) 28195719

[22] Schulze TP. 2002 Kinetic Monte Carlo simulations with minimal searching. Phys. Rev. E. **65**, 036704. (10.1103/Physreve.65.036704) 11909304

[23] Chatterjee A, Vlachos DG. 2007 An overview of spatial microscopic and accelerated kinetic Monte Carlo methods. J. Comput. Aided Mater. Des. **14**, 253-308. (10.1007/s10820-006-9042-9)

[24] Hess F. 2019 Efficient implementation of cluster expansion models in surface kinetic Monte Carlo simulations with lateral interactions: subtraction schemes, supersites, and the supercluster contraction. J. Comput. Chem. **40**, 2664-2676. (10.1002/jcc.26041) 31418885

[25] Nettesheim S, Vonoertzen A, Rotermund HH, Ertl G. 1993 Reaction-diffusion patterns in the catalytic CO oxidation on Pt(110) - front propagation and spiral waves. J. Chem. Phys. **98**, 9977-9985. (10.1063/1.464323)

[26] Kapička J, Marek M. 1989 Oscillations on individual catalytic pellets in a packed-bed - CO oxidation on Pt/Al_2_O_3_. J. Catal. **119**, 508-511. (10.1016/0021-9517(89)90178-4)

[27] Kapička J, Marek M. 1989 Transition to chaos in the oscillating CO oxidation on Pt/Al_2_O_3_. Surf. Sci. **222**, L885-L889. (10.1016/0039-6028(89)90359-2)

[28] Marek M, Schejbal M, Kočí P, Nevoral V, Kubicek M, Hadac O, Schreiber I. 2006 Oscillations, period doublings, and chaos in CO oxidation and catalytic mufflers. Chaos. **16**, 037107. (10.1063/1.2354429) 17014241

[29] Stotzel J, Frahm R, Kimmerle B, Nachtegaal M, Grunwaldt JD. 2012 Oscillatory behavior during the catalytic partial oxidation of methane: following dynamic structural changes of palladium using the QEXAFS technique. J. Phys. Chem. C. **116**, 599-609. (10.1021/jp2052294)

[30] Ciambelli P, Di Benedetto A, Garufi E, Pirone R, Russo G. 1998 Spontaneous isothermal oscillations in N_2_O decomposition over a Cu-ZSM5 catalyst. J. Catal. **175**, 161-169. (10.1006/jcat.1998.1986)

[31] Janssen NMH, Cobden PD, Nieuwenhuys BE, Ikai M, Mukai K, Tanaka K. 1995 Hysteresis and oscillations in the selectivity during the NO-H_2_ reaction over Rh(533). Catal. Lett. **35**, 155-163. (10.1007/Bf00807013)

[32] Imbihl R. 1993 Oscillatory reactions on single-crystal surfaces. Prog. Surf. Sci. **44**, 185-343. (10.1016/0079-6816(93)90086-B)

[33] Schüth F, Henry BE, Schmidt LD. 1993 Oscillatory reactions in heterogeneous catalysis. Advances in Catalysis. **39**, 51-127. (10.1016/S0360-0564(08)60577-5)

[34] Belousov BP. 1959 Periodicheski deistvuyushchaya reaktsia i ee mekhanism [Periodically acting reaction and its mechanism]. sbornik referatov po radiotsionnoi meditsine [collection of abstracts on radiation medicine], pp. 145-147. Moscow: Medgiz.

[35] Chatzinikolaou TP, Fyrigos IA, Ntinas V, Kitsios S, Tsompanas MA, Bousoulas P, Tsoukalas D, Adamatzky A, Sirakoulis GC. 2022 Chemical Wave Computing from Labware to Electrical Systems. Electronics-Switz. **11**, 1683. (10.3390/electronics11111683)

[36] Mikhailov AS, Showalter K. 2006 Control of waves, patterns and turbulence in chemical systems. Phys. Rep. **425**, 79-194. (10.1016/j.physrep.2005.11.003)

[37] Jefferson DR. 1985 Virtual Time. ACM Trans. Program. Lang. Syst. **7**, 404-425. (10.1145/3916.3988)

[38] Lubachevsky BD. 1988 Efficient parallel simulations of dynamic Ising spin systems. J. Comput. Phys. **75**, 103-122. (10.1016/0021-9991(88)90101-5)

[39] Shim Y, Amar JG. 2005 Semirigorous synchronous sublattice algorithm for parallel kinetic Monte Carlo simulations of thin film growth. Phys. Rev. B. **71**, 125432. (10.1103/PhysRevB.71.125432)

[40] Merrick M, Fichthorn KA. 2007 Synchronous relaxation algorithm for parallel kinetic Monte Carlo simulations of thin film growth. Phys. Rev. E. **75**, 011606. (10.1103/Physreve.75.011606) 17358166

[41] Martínez E, Marian J, Kalos MH, Perlado JM. 2008 Synchronous parallel kinetic Monte Carlo for continuum diffusion-reaction systems. J. Comput. Phys. **227**, 3804-3823. (10.1016/j.jcp.2007.11.045)

[42] Arampatzis G, Katsoulakis MA, Plechac P, Taufer M, Xu LF. 2012 Hierarchical fractional-step approximations and parallel kinetic Monte Carlo algorithms. J. Comput. Phys. **231**, 7795-7814. (10.1016/j.jcp.2012.07.017)

[43] Oppelstrup T, Jefferson DR, Bulatov VV, Zepeda-Ruiz LA. 2016 SPOCK: Exact parallel kinetic Monte-Carlo on 1.5 million tasks. In *Proc. of the 2016 ACM SIGSIM Conf. on Principles of Advanced Discrete Simulation (SIGSIM-PADS '16)*, *Banff, Alberta, Canada*, *May 15–18, 2016*. New York, NY: Association for Computing Machinery (ACM).

[44] Piccinin S, Stamatakis M. 2017 Steady-state CO oxidation on Pd(111): first-principles kinetic Monte Carlo simulations and microkinetic analysis. Top. Catal. **60**, 141-151. (10.1007/s11244-016-0725-5)

[45] Glansdorff P, Prigogine I. 1971 Thermodynamics of structure, stability and fluctuations. New York, NY: Wiley-Interscience.

[46] Prigogine I, Lefever R. 1968 Symmetry breaking instabilities in dissipative systems. II. J. Chem. Phys. **48**, 1695-1700. (10.1063/1.1668896)

[47] Nicolis G, Prigogine I. 1977 Self-organization in nonequilibrium systems: from dissipative structures to order through fluctuations. New York, NY: Wiley.

[48] Wellner M, Berenfeld O. 2004 Chapter 35 - theory of reentry. In Cardiac electrophysiology (eds DP Zipes, J Jalife), pp. 317-326, 4th edn. Philadelphia, PA: W. B. Saunders.

[49] Kolakowska A, Novotny MA. 2005 Desynchronization and speedup in an asynchronous conservative parallel update protocol. In Artificial intelligence and computer science(ed. S Shannon), pp. 151-176. New York, NY: Nova Science Publishers, Inc.

[50] Korniss G, Novotny MA, Guclu H, Toroczkai Z, Rikvold PA. 2003 Suppressing roughness of virtual times in parallel discrete-event simulations. Science **299**, 677-679. (10.1126/science.1079382) 12560543

[51] Shchur LN, Shchur LV. 2015 Relation of parallel discrete event simulation algorithms with physical models. J. Phys: Conf. Ser. **640**, 012065. (10.1088/1742-6596/640/1/012065)

[52] Shchur LN, Novotny MA. 2004 Evolution of time horizons in parallel and grid simulations. Phys. Rev. E **70**, 026703. (10.1103/PhysRevE.70.026703) 15447616

[53] Savva GD, Benson RL, Christidi I-A, Stamatakis M. 2023 Exact distributed kinetic Monte Carlo simulations for on-lattice chemical kinetics: lessons learnt from medium- and large-scale benchmarks. Figshare. (10.6084/m9.figshare.c.6486293) PMC10200346 37211035

